# Factors associated with single and multiple suicide attempts in adolescents attending school in Argentina: national cross-sectional survey in 2018

**DOI:** 10.1192/bjo.2022.524

**Published:** 2022-07-07

**Authors:** Supa Pengpid, Karl Peltzer

**Affiliations:** Department of Health Education and Behavioral Sciences, Faculty of Public Health, Mahidol University, Bangkok, Thailand; and Department of Research Administration and Development, University of Limpopo, Turfloop, South Africa; Department of Psychology, University of the Free State, Bloemfontein, South Africa; and Department of Psychology, College of Medical and Health Science, Asia University, Taichung, Taiwan

**Keywords:** Multiple suicide attempts, risk factors, adolescents, Argentina

## Abstract

**Background:**

Factors associated with single suicide attempts (SSA) and multiple suicide attempts (MSA) may differ.

**Aims:**

The study aimed to assess the factors associated with MSA in adolescents with a history of suicide attempts during the past 12 months in Argentina.

**Method:**

National cross-sectional data from the Global School-based Student Health Survey in Argentina in 2018 were analysed. Students who reported having a history of suicide attempts in the past 12 months were included in the final sample (*n* = 8507). Students with MSA were compared with students with an SSA through multiple logistic regression.

**Results:**

In a subsample of adolescents attending school (mean age 14.8 years, s.d. = 1.3), 59.4% had an SSA and 40.6% had MSA in the past 12 months. In the final adjusted logistic regression model, compared with participants with SSA, both male and female students with MSA more frequently had no close friends, reported feeling more lonely and had more anxiety-induced sleep disturbances. Furthermore, among female participants, having been physically attacked, having participated in physical fights, low parental support, current tobacco use and lifetime amphetamine use were associated with MSA. Among male students, multiple sexual partners were associated with MSA. Furthermore, among both boys and girls, compared with participants without psychosocial distress, participants with one, two, three or more psychosocial distress factors had higher odds of MSA. Compared with students with one or two social or environmental risk factors, students with seven or eight social or environmental risk factors had higher odds of MSA; compared with students who had zero or one health risk behaviours, students with six or more health risk behaviours had higher odds of MSA.

**Conclusions:**

Psychosocial distress (anxiety-induced sleep disturbance, having no close friends and loneliness) increased the odds of MSA among both sexes. The odds of MSA were increased by interpersonal violence, low parental support and substance use among girls, and by having multiple sexual partners among boys. This suggests the potential relevance of these variables in identifying multiple suicide attempters among adolescents attending school in Argentina.

Suicide constitutes a major killer among youth.^1^ Suicide attempts are risk factors for subsequent suicide attempts, and repeated suicide attempts further increase the risk of both additional suicide attempts and suicide.^2^ Little research has been done among adolescents on the differences between those with a single suicide attempt (SSA) and those with multiple suicide attempts (MSA), especially in Latin America.^2,3^ SSA is an important predictor of MSA, because in most studies 16–34% of subjects have a subsequent suicide attempt within the first 1–2 years after their initial suicide attempt.^4^ Therefore, it is of the utmost relevance to determine which factors are associated with MSA.^2,3^ It has been theorised that the experience of suicide attempt increases the susceptibility of the individual to both MSA and suicide.^5,6^ This may be because the barrier or taboo against suicide is removed after the initial suicide attempt; thus, individuals may perceive suicide as a more viable option when stressors arise.^6^

Based on previous research,^7^ risk factors for MSA were conceptualised into psychosocial distress factors, negative social or environmental factors, and health-compromising behaviours. Some research comparing SSA and MSA among adolescents in USA and Australia found that psychosocial distress factors (depression,^8,9^ comorbid health risks,^9^ being a victim of physical assault^9^ and history of sexual abuse^10^), negative social–environmental factors (lower social support of family, friends or non-family adults,^11^ and lack of mental healthcare following the first suicide attempt^12^) and health risk behaviours (externalising disorder,^13^ sexual risk behaviour,^9^ substance use^9,10,13^ and serious self-mutilation^14^) were associated with MSA. However, most of these investigations had a selection bias owing to only using in-patient or emergency room department samples. Therefore, their results cannot be generalised to the general community,^2^ and no such studies have been conducted in Latin America. Developmental trends may also influence the transition from SSA to MSA. For example, in a 90-country study, younger adolescents attending school (13–15 years old) had different associations with suicide attempt than older adolescents attending school (16–17 years old).^15^

## Prevalence

In Latin America, the prevalence of suicide attempt in the past 12 months among adolescents attending school (13–15 years old) was 17.9% in Andean countries, followed by 15.7% in Southern cone countries, including Argentina, and 13.2% in Central American countries.^16^ Current alcohol use and lack of peer support increased the risk of suicide attempt across the subregions in Latin America.^16^ In a study among adolescents attending school in Bolivia, Costa Rica, Honduras, Peru and Uruguay, bullying victimisation was highly associated with suicide attempt.^17^ Suicide mortality rates in the region of the Americas from 2001 to 2008 showed an increase among young people in five countries, including Argentina.^18–20^ In Argentina, rates of suicidal ideation and suicide planning significantly increased among girls but not among boys from 2007 to 2018; this could be in part attributed to a higher decline in parental support among girls.^21^ In the 2012 Global School-based Student Health Survey (GSHS) in Argentina, parental support was found to be protective against suicide attempts.^22^ In different studies among youths admitted to hospital in Argentina, major precedents of suicide attempts were previous attempted suicides,^23,24^ behavioural and conduct disorders,^23–25^ depression,^25^ labile emotional balance and exacerbated impulsiveness,^26^ and family disorders (family structure and functioning, single-parent family and family relationships).^24,26^ In an investigation among adults with suicide attempts in a public hospital in Argentina, poor family functioning was identified as a risk factor for suicide attempt.^27^

## Aims and objectives

Considering this background, the present study tried to fill the identified gap by researching the differences between adolescents with MSA versus SSA using a large school-based sample from Argentina. By examining sociodemographic factors,^11,28^ psychosocial distress factors including multiple adverse experiences,^7,28–31^ negative social or environmental factors (such as low parental or peer support)^11,28^ and health-compromising behaviours (tobacco use,^28^ bullying victimisation,^32^ and soft drink^33^ and fast food^34^ intake), we sought to identify relevant factors that may affect the relationship between MSA and SSA in the general adolescent population in Argentina.

## Method

### Sample

Publicly available data from the nationally representative cross-sectional 2018 Argentina GSHS were analyzed.^35^ More details on the study and the data are publicly available on the World Health Organization website.^35^ Briefly, the main objective of the GSHS was to measure the risk and protective factors of the main non-communicable diseases.

### Procedure

A two-stage cluster sample design was used to generate a representative sample of all students in the eighth grade of primary school/polymodal or first year of high school to third year/12th grade polymodal or fifth year of high school in Argentina (age range ≤11 to ≥18 years). At the initial stage, schools were selected with a probability proportional to the size of the enrolment. At the subsequent stage, classes were randomly selected and all students in selected classes were eligible to participate, regardless of age.^35^ Students completed a self-administered questionnaire in Spanish under the supervision of trained external survey administrators.^35^ The school response rate was 86%, the student response rate was 74% and the overall response rate was 63%.^35^ From the total sample of 56 981, we restricted our analyses to those who had a history of suicide attempts during the past 12 months (*n* = 8507).

The authors assert that all procedures contributing to this work comply with the ethical standards of the relevant national and institutional committees on human experimentation and with the Helsinki Declaration of 1975, as revised in 2008. All procedures involving human subjects were approved by the ethics committee of the Ministerio de Salud y Desarrollo Social de la Nación, and written informed consent was obtained from the participating schools, parents and students.^35^

### Measures

The GSHS questions used are shown in Table 1.^35^ The GSHS is a sister study of the US ‘Youth Risk Behavior Survey’ for which test–retest reliability has been proven.^36^ Moreover, the GSHS questionnaire showed a test–retest agreement of 77% and a Cohen's kappa of 0.47.^37^

**Table 1:** Questionnaire items used in this survey

Variable name	Item description	Response options (coding scheme)
Suicide attempt	‘During the past 12 months, how many times did you actually attempt suicide?’	‘1 = 0 times to 5 = 6 or more times (coded 1 = 1, 2 = 2 and 3–5 = 3)’
Age	‘How old are you?’	‘11 years old or younger to 18 years old or older’
Sex	‘What is your sex?’	‘Male, Female’
	*Psychosocial distress*	
No close friends	‘How many close friends do you have?’	‘1 = 0 to 4 = 3 or more (coded 1+=0, 0 = 1)’
Loneliness	‘During the past 12 months, how often have you felt lonely?’	‘1 = never to 5 = always (coded 1–3 = 0 and 4–5 = 1)’
Anxiety-induced sleep disturbance	‘During the past 12 months, how often have you been so worried about something that you could not sleep at night?’	‘1 = never to 5 = always (coded 1–3 = 0 and 4–5 = 1)’
Traditional and cyberbullied	‘[Bullying occurs when one or more students or someone else about your age teases, threatens, ignores, spreads rumours about, hits, shoves, or hurts another person over and over again. It is not bullying when two people of about the same strength or power argue or fight or tease each other in a friendly way.]’	
	1) ‘During the past 12 months, have you ever been bullied on school property?’	'1 = Yes, 0 = No'
	2) ‘During the past 12 months, have you ever been bullied when you were not on school property?’	'1 = Yes, 0 = No'
	3) ‘During the past 12 months, have you ever been cyber bullied? (Count being bullied through texting, Instagram, Snapchat, Facebook, WhatsApp, Edmodo, Messenger, or other social media.)’	'1 = Yes, 0 = No (coded 1 or 2 = 1 and 3 = 1)'
Physically attacked	‘During the past 12 months, how many times were you physically attacked?’	‘1 = 0 times to 8 = 12 or more times (coded 1–2 = 0 and 3–8 = 1)’
Physical fights	‘During the past 12 months, how many times were you in a physical fight?’	‘1 = 0 times to 8 = 12 or more times (coded 1–2 = 0 and 3–8 = 1)’
	*Social–environmental factors*	
Hunger	‘During the past 30 days, how often did you go hungry because there was not enough food in your home?’	‘1 = never to 5 = always (coded 1–3 = 0 and 4–5 = 1)’
Low peer support	‘During the past 30 days, how often were most of the students in your school kind and helpful?’	‘1 = never to 5 = always (coded 1–2 = 1, 3–5 = 0)’
Low parental supervision	‘During the past 30 days, how often did your parents or guardians check to see if your homework was done?’	‘1 = never to 5 = always (coded 1–2 = 1 and 3–5 = 0)’
Low parental connectedness	‘During the past 30 days, how often did your parents or guardians understand your problems and worries?’	‘1 = never to 5 = always (coded 1–2 = 1 and 3–5 = 0)’
Low parental bonding	‘During the past 30 days, how often did your parents or guardians really know what you were doing with your free time?’	‘1 = never to 5 = always (coded 1–2 = 1 and 3–5 = 0)’
Low parental respect for privacy	‘During the past 30 days, how often did your parents or guardians go through your things without your approval?’	‘1 = never to 5 = always (coded 1–3 = 0 and 4–5 = 1)’
Parental tobacco use	‘Which of your parents or guardians use any form of tobacco?’	‘1 = neither to 4 = both (coded 1 = 0 and 2–4 = 1)’
Passive smoking	‘During the past 7 days, on how many days have people smoked in your presence?’	‘1 = 0 days to 5 = all 7 days’ ‘(coded 1 = 0 and 2–5 = 1)’
School truancy	‘During the past 30 days, on how many days did you miss classes or school without permission?’	‘1 = 0 days to 5 = 10 or more days (coded 1–2 = 0 and 3–5 = 1)’
	*Health risk behaviour*	
Current cannabis use	‘During the past 30 days, how many times have you used marijuana?’	‘1 = 0 times to 5 = 20 or more times (coded 1 = 0 and 2–5 = 1)’
Current tobacco use	‘During the past 30 days, on how many days did you smoke cigarettes/use any tobacco products other than cigarettes, such as pipes, narguile, or smokeless tobacco?’	‘1 = 0 days to 7 = all 30 days (coded 1 = 0 and 2–7 = 1)’
Current alcohol use	‘During the past 30 days, on how many days did you have at least one drink containing alcohol?’	‘1 = 0 days to 7 = all 30 days’ ‘(coded 1 = 0 and 2–7 = 1)’
Amphetamine use	‘During your life, how many times have you used amphetamines or methamphetamines?’	‘1 = 0 times to 5 = 20 or more times (coded 1 = 0 and 2–5 = 1)’
Multiple sexual partners	‘During your life, with how many people have you had sexual intercourse?’	‘1 = never had sex to 7 = 6 or more people (coded 1 = 3–7 and 0 = 1–2)’
Soft drink intake	‘During the past 30 days, how many times per day did you usually drink carbonated soft drinks, such as Coca-Cola or Fanta? (Do not include diet soft drinks.)’	‘1 = 0 times to 7 = 5 or more times (coded 1–2 = 0 and 3–7, 2 or more times)’
Fast food intake	‘During the past 7 days, on how many days did you eat food from a fast food restaurant, such as Mahelaba, Snack, pizzeria, McDonald's?’	‘1 = 0 days to 8 = 7 days (coded 1–2 = 0 and 3–6 = 1, 2 or more days)’
Injury	‘During the past 12 months, how many times were you seriously injured?’	‘1 = 0 times to 8 = 12 or more times (coded 1 = 0 and 2–8 = 1)’

The outcome variable ‘suicide attempts’ was evaluated with the question ‘During the past 12 months, how many times did you actually attempt suicide?’^35^

Six items were used to assess psychosocial distress: having no close friends, loneliness (mostly or always), anxiety-induced sleep disturbance (mostly or always), bullied (≥1 or 2 days/month), physically attacked (≥1 times/year) and involvement in physical fighting (≥1 times/year). Seven questions were used to assess negative social or environmental factors: experiencing hunger (mostly or always), low (never or rarely) support by peers, parental tobacco use, passive smoking (≥1 day/week), school truancy (≥1 or 2 days/month) and low parental support (never or rarely parental/guardian checking of home work, understanding of problems and worries, ‘really knowing what you were doing with your free time when you were not at school or work,’ and parents/guardians mostly/always go through things. The four parental support items were summed and grouped as high (0–1), moderate (2) and lowest (3–4) levels of parental support (as in previous studies^28^). Eight items were used to assess health-compromising behaviours: current use of tobacco, alcohol and cannabis; fast food intake (≥1 day/week); lifetime use of amphetamines; multiple sexual partners (≥2 sexual partners in lifetime); consumption of soft drinks (≥1/day) and physical injury (≥1/year).

### Statistical analysis

STATA software version 15.0 (Stata Corporation, College Station, Texas, USA) was used for statistical analyses. Data were weighted for the probability selected and non-response. To test differences in proportions, Pearson's χ^2^-tests were used. Adjusted logistic regression analyses were applied to estimate independent predictors of MSA versus SSA. History of suicide attempts in the past 12 months were coded here as 1 (two or more times) and 0 (one time). Unadjusted and adjusted logistic regression analyses were used to estimate predictors of MSA versus SSA. The multivariable logistic regression model was adjusted for sociodemographic variables, psychosocial distress factors, negative social or environmental factors, and health-compromising behaviours. Moreover, adjusted logistic regression analyses were applied to estimate associations between the number of psychosocial distress factors, negative social or environmental factors, health-compromising behaviours and MSA versus SSA. This multivariable logistic regression model was adjusted for sociodemographic variables, number of psychosocial distress factors, number of negative social or environmental factors, and number of health-compromising behaviours. Missing values (<1.9% for suicide attempts and <4.3% for all other variables) were excluded, and *P* < 0.05 was accepted as significant.

## Results

### Sample and suicide attempt characteristics

The subsample consisted of 8507 adolescents attending school (mean age 14.8 years, s.d. = 1.3) who had either an SSA (*n* = 5105, 69.4%) or MSA (*n* = 3402, 40.6%) during the past 12 months. The majority of the sample (65.9%) was female. Almost one in ten students (9.3%) had no close friends, 32.1% had anxiety-induced sleep disturbance, 35.3% had been attacked, 41.4% were lonely, 27.2% had often been traditionally and cyberbullied and 38.5% had been involved in physical fighting. More than one-third of the students (38.0%) were current tobacco users, 17.3% used cannabis currently, 8.1% had ever used amphetamine, 39.5% had parents who used tobacco, 38.2% consumed soft drinks (≥1 times/day), 42.3% had fast food (≥1 days/week) and 46.1% had a serious physical injury (≥1 times/year). Male students had a lower rate of MSA than female students. When comparing SSA with MSA, the proportions of all psychosocial distress variables, all negative social–environmental factors and six of eight health risk behaviours were higher in students with MSA. Further sample details are shown in Table 2.

**Table 2:** Sample characteristics and odds ratios of single and multiple suicide attempters among adolescents attending school in Argentina

Variable	Sample	Single attempt	Multiple attempts	Odds ratio (95% CI)
	*N* = 8507	*N* = 5105	*N* = 3402	
**Sociodemographics**	%	%	%	
All		59.4	40.6	
Age (years)				
≤13	20.9	20.7	21.1	1 (Reference)
14	25.1	25.9	24.0	1.01 (0.88, 1.15)
15	23.2	22.6	24.0	1.09 (0.95, 1.24)
≥16	30.8	30.7	30.9	0.94 (0.83, 1.06)
Gender				
Female	65.9	61.2	73.0	1 (Reference)
Male	34.1	38.8	27.0	0.62 (0.57, 0.68)***
**Psychosocial distress**				
Having no close friends	9.3	8.3	10.9	1.51 (1.31, 1.75)***
Loneliness	41.4	32.5	54.5	2.38 (2.17, 2.60)***
Anxiety-induced sleep disturbance	32.1	24.5	43.1	2.10 (1.91, 2.31)***
Traditional and cyberbullied	27.2	22.6	34.1	1.56 (1.42, 1.73)***
Physically attacked	35.3	30.1	43.0	1.70 (1.55, 1.86)***
Physical fight	38.5	34.4	44.6	1.46 (1.34, 1.61)***
**Negative social–environmental factors**				
Feeling hungry (mostly or always)	5.2	3.8	7.4	2.02 (1.65, 2.49)***
Peer support (low)	39.8	37.9	42.7	1.31 (1.20, 1.43)***
Parental support				
High	39.1	42.2	34.5	1 (Reference)
Moderate	26.9	27.8	25.5	1.21 (1.08, 1.36)***
Lowest	34.0	30.0	40.0	1.80 (1.62, 2.00)***
Parental tobacco use	39.5	37.1	43.0	1.25 (1.15, 1.37)***
Passive smoking	75.7	71.7	81.5	1.40 (1.26, 1.56)***
School truancy	39.6	35.7	45.4	1.30 (1.19, 1.42)***
**Health risk behaviours**				
Current tobacco use	38.0	30.9	48.5	1.75 (1.60, 1.91)***
Current alcohol use	67.2	63.1	73.2	1.37 (1.25, 1.51)***
Current cannabis use	17.3	14.4	21.8	1.76 (1.57, 1.97)***
Lifetime amphetamine use	8.1	6.1	11.2	2.25 (1.91, 2.66)***
Multiple sexual partners	29.6	24.7	36.8	1.43 (1.30, 1.57)***
Soft drink intake (≥1 times/day)	38.2	36.1	41.4	1.09 (0.99, 1.19)
Fast food intake (≥1 days/week)	42.3	42.4	42.2	0.99 (0.91, 1.08)
Physical injury	46.1	42.7	51.1	1.27 (1.16, 1.40)***

*P < 0.05; **P < 0.01; ***P < 0.001.

### SSA and MSA among adolescents attending school by sex in Argentina

Of the six psychosocial distress variables evaluated, all were higher in individuals with MSA than in those with SSA in both boys and girls. Of the eight health risk behaviour variables assessed, all were higher in MSA than in SSA in boys, and, among girls, six health risk behaviour variables were higher in MSA than in SSA. Of the six negative social–environmental factors measured, among males, five were higher in MSA than in SSA and, among females, four were higher in MSA than in SSA (Table 3).

**Table 3:** Single and multiple suicide attempts among adolescents attending school by sex in Argentina

Variable	Male				Female			
	Sample	Single attempt	Multiple attempts	*P*-value	Sample	Single attempt	Multiple attempts	*P*-value
	*N* = 2831	*N* = 1911	*N* = 920		*N* = 5543	*N* = 3126	*N* = 2417	
**Sociodemographics**	%	%	%		%	%	%	
Age (years)								
≤13	18.6	19.7	16.1	0.634	22.0	21.5	22.7	0.554
14	24.6	24.3	25.2		25.3	26.5	23.8	
15	22.5	22.8	21.9		23.7	22.6	25.0	
≥16	34.3	34.1	36.8		29.0	29.4	28.5	
**Psychosocial distress**								
Having no close friends	10.7	9.5	13.5	<0.001	8.5	7.3	10.0	<0.001
Loneliness	30.5	23.0	46.5	<0.001	46.9	38.2	57.7	<0.001
Anxiety-induced sleep disturbance	21.7	15.7	34.4	<0.001	37.1	29.8	46.1	<0.001
Traditional and cyberbullied	19.2	16.1	26.3	<0.001	31.0	26.7	36.2	0.002
Physically attacked	38.4	34.3	47.3	<0.001	33.4	27.6	40.7	<0.001
Physical fight	50.4	46.4	59.2	0.002	32.2	26.8	38.9	<0.001
**Negative social–environmental factors**								
Feeling hungry (mostly or always)	6.8	5.2	10.4	<0.001	4.4	2.9	6.3	<0.001
Peer support (low)	38.6	36.1	44.0	<0.001	40.4	38.9	42.3	0.239
Parental support								
High	42.8	44.6	38.6	0.135	37.2	40.5	33.0	<0.001
Moderate	27.1	27.6	25.8		26.9	28.2	25.3	
Lowest	30.1	27.8	35.5		36.0	31.3	41.7	
Parental tobacco use	39.1	36.2	45.6	0.022	39.6	37.7	42.0	0.111
Passive smoking	72.7	69.4	79.6	0.003	77.3	73.3	82.2	<0.001
School truancy	42.5	39.7	48.6	0.035	38.0	33.1	44.0	<0.001
**Health risk behaviours**								
Current tobacco use	35.0	28.9	47.9	<0.001	39.3	32.1	48.5	<0.001
Current alcohol use	62.3	58.2	71.5	<0.001	69.5	66.3	73.5	0.005
Current cannabis use	20.9	17.1	29.3	<0.001	15.3	12.6	18.7	0.004
Lifetime amphetamine use	12.2	9.5	18.2	<0.001	6.0	3.9	8.7	<0.001
Multiple sexual partners	39.2	35.7	47.7	<0.001	24.5	19.9	30.3	<0.001
Soft drink intake (≥1 times/day)	38.0	35.0	44.3	0.021	38.3	36.5	40.5	0.144
Fast food intake (≥1 days/week)	42.8	40.1	48.5	0.037	42.1	43.7	40.0	0.174
Physical injury	52.1	47.2	62.1	<0.001	42.5	39.4	46.3	0.017

### Association between single risk factors and MSA

In the final adjusted logistic regression model, compared with participants with SSA, both male and female students with MSA more frequently had no close friends (adjusted odds ratio [AOR]: 1.50, 95% CI: 1.00–2.26 among boys and AOR: 1.52, 95% CI: 1.16–1.56 among girls), reported feeling more lonely (AOR: 2.56, CI: 1.99–3.31 among boys and AOR: 1.74, 95% CI: 1.51–2.02 among girls) and had more anxiety-induced sleep disturbances (AOR: 1.57, CI: 1.20–2.07 among boys and AOR: 1.35, 95% CI: 1.16–1.57 among girls). Furthermore, among female participants, having been physically attacked (AOR: 1.26, 95% CI: 1.08–1.47), having participated in physical fights (AOR: 1.33, 95% CI: 1.13–1.57), having low parental support (AOR: 1.45, 95% CI: 1.23–1.71), current tobacco use (AOR: 1.20, 95% CI: 1.01–1.41) and use of amphetamines (AOR: 1.47, 95% CI: 1.04–2.07) were associated with MSA. Among male students, having multiple sexual partners (AOR: 1.30, 95% CI: 1.01–1.67) was associated with MSA (Table 4).

**Table 4:** Associations between single and multiple suicide attempts by single risk factors

	Male	Female
Variable	AOR (95% CI)^a^^,^^b^	AOR (95% CI)^a^^,^^c^
**Psychosocial distress**		
Having no close friends	1.50 (1.00, 2.26)*	1.52 (1.16, 1.56)***
Loneliness	2.56 (1.99, 3.31)***	1.74 (1.51, 2.02)***
Anxiety-induced sleep disturbance	1.57 (1.20, 2.07)***	1.35 (1.16, 1.57)***
Traditional and cyberbullied	1.25 (0.94, 1.67)	0.94 (0.87, 1.16)
Physically attacked	1.10 (0.87, 2.49)	1.26 (1.08, 1.47)**
Physical fight	1.16 (0.91, 1.48)	1.33 (1.13, 1.57)***
**Negative social–environmental factors**		
Feeling hungry (mostly or always)	1.47 (0.87, 2.49)	1.42 (0.99, 2.04)
Peer support (low)	1.02 (0.81, 1.30)	0.93 (0.80, 1.07)
Parental support		
High	1 (Reference)	1 (Reference)
Moderate	0.93 (0.71, 1.24)	1.05 (0.88, 1.25)
Lowest	1.11 (0.84, 1.46)	1.45 (1.23, 1.71)***
Parental tobacco use	1.05 (0.83, 1.33)	1.00 (0.87, 1.16)
Passive smoking	1.05 (0.79, 1.38)	1.08 (0.90, 1.29)
School truancy	1.01 (0.80, 1.28)	1.09 (0.94, 1.26)
**Health risk behaviours**		
Current tobacco use	1.32 (0.99, 1.77)	1.20 (1.01, 1.41)*
Current alcohol use	0.94 (0.72, 1.23)	0.94 (0.80, 1.10)
Current cannabis use	1.01 (0.71, 1.43)	1.06 (0.84, 1.35)
Ever amphetamines	1.54 (0.93, 2.53)	1.47 (1.04, 2.07)*
Multiple sexual partners	1.30 (1.01, 1.67)*	1.16 (0.98, 1.38)
Physical injury	1.12 (0.89, 1.41)	1.05 (0.91, 1.20)

a Adjusted for sociodemographic factors, and all variables in the table.

b Log likelihood (LL) = 95 460.87, Nagelkerke R^2^ = 0.23.

c LL = 237 667.98, Nagelkerke R^2^ = 0.13.

*P < 0.05; **P < 0.01; ***P < 0.001.

### Association between multiple risk factors and MSA

In the final adjusted logistic regression model, among both sexes, compared with participants without psychosocial distress, participants with one, two, three or more psychosocial distress factors had higher odds of MSA. Compared with students with one or two social or environmental risk factors, students with seven or eight social or environmental risk factors had a higher odds of MSA, and compared with students who had zero or one health risk behaviours, students with six or more health risk behaviours had higher odds of MSA (Table 5).

**Table 5:** Associations between single and multiple suicide attempts by multiple risk factors

Number of risk factors	Male		Female	
	Prevalence (%)	AOR (95% CI)^a^^,^^b^	Prevalence (%)	AOR (95% CI)^a^^,^^c^
**Psychosocial distress factors**				
0	22.6	1 (Reference)	20.2	1 (Reference)
1	29.2	2.05 (1.43, 2.93)***	23.5	1.79 (1.45, 2.22)***
2	22.5	2.57 (1.78, 3.71)***	23.8	2.15 (1.73, 2.66)***
3 or more	25.7	5.31 (3.68, 7.67)***	32.5	3.02 (2.44, 3.72)***
**Negative social or environmental factors**				
1–2	21.3	1 (Reference)	17.2	1 (Reference)
3–4	47.7	1.19 (0.89, 1.60)	46.2	1.11 (0.91, 1.35)
5–6	25.0	1.31 (0.93, 1.84)	31.7	1.49 (1.21, 1.84)***
7–8	6.0	1.91 (1.06, 3.46)*	4.8	2.20 (1.50, 3.23)***
**Health-compromising behaviours**				
0–1	27.3	1 (Reference)	26.9	1 (Reference)
2–3	39.3	1.46 (1.09, 1.96)*	41.9	0.94 (0.79, 1.11)
4–5	23.5	1.28 (0.91, 1.79)	24.6	1.11 (0.91, 1.35)
6–8	10.0	1.60 (1.04, 2.48)*	6.7	1.61 (1.19, 2.19)**

a. Adjusted for sociodemographic factors, and all variables in the table.

b. LL = 88 766.15, Nagelkerke R^2^ = 0.18.

c. LL = 227 797.29, Nagelkerke R^2^ = 0.11.

*P < 0.05; **P < 0.01; ***P < 0.001.

## Discussion

This investigation aimed to estimate psychosocial distress factors, social or environmental factors, and health risk behaviour correlates of MSA versus SSA in adolescents attending school in Argentina. The findings related to psychosocial distress, low parental support and health-compromising behaviour variables associated with MSA versus SSA were largely consistent with those of previous research.^8–11,13^ Having no close friends, loneliness, and anxiety-induced sleep disturbance, among both boys and girls, were able to differentiate between adolescents with MSA versus SSA. Having been physically attacked, having participated in physical fights, low parental support, current tobacco use and lifetime amphetamine use were able to differentiate between girls with MSA versus SSA. Having had multiple sexual partners was able to differentiate between boys with MSA versus SSA.

Several studies^2,4,17,31^ have shown that having a mental disorder, hopelessness and stressful life events such as interpersonal violence predict MSA. Echoing these findings, the treatment of psychosocial distress factors and reduction of interpersonal violence, including being bullied, are important in the prevention of MSA. In line with some previous research,^12,38,39^ we found that female sex and lower socioeconomic status (using food insecurity as a proxy) increased the odds of MSA. However, contrary to some research,^12^ we did not find significant age differences in relation to the prevalence of MSA versus SSA. A previous study among adolescents^12^ that assessed lifetime suicide attempts found that younger age of first suicide attempt was associated with MSA; by contrast, our study assessed only suicide attempts during the past 12 months, which may explain the non-significant age differences in MSA in this study. The preponderance of MSA among girls appeared to be consistent with a previous systematic review showing that females (12–26 years) had a higher risk of suicide attempt.^39^

Consistent with previous studies,^7,29,30^ this investigation demonstrated that MSA increased with increases in multiple risk factors, possibly confirming a dose–response relationship. We found an association between anxiety-induced sleep disturbance and MSA. Similarly, in a previous study^3^ among adults, an association was found between the frequency of nightmares and MSA. Knowledge of the variables that are potentially associated with subsequent suicide attempts can be helpful to healthcare providers, who could detect anxiety-induced sleep disturbance, lack of close friends and loneliness to identify MSA risk. Asking about anxiety-induced sleep problems and loneliness may be less threatening than asking about suicidal ideation or intent.^3^ Furthermore, low parental support was among girls associated with MSA in this study. In the 2012 GSHS in Argentina, parental support was found to be protective against suicide attempts.^22^ According to a previous trend study among adolescent girls in Argentina, parental support decreased from 2007 to 2018.^21^ The reduction in parental support could be attributed to changes in the family system in Argentina.^40^ Previous studies among adolescents in Latin America^16,17^ found associations of suicide attempt with current alcohol use, lack of peer support and bullying victimisation, whereas we did not find such associations with MSA in our study.

The limitations of this study include the inclusion of only adolescents attending school, the cross-sectional survey design and the assessment by self-report. Moreover, the GSHS in Argentina did not assess suicide attempts prior to the past 12 months, suicide attempt methods, family history of suicide or help-seeking behaviours for suicidal behaviours. Future studies should assess these variables in order to more comprehensively measure factors differentiating SSA and MSA. Moreover, several concepts were only assessed with single-item questions, which are limited; future research should include full scales, such as those on depression or childhood adverse events. Comparing the demographics of this subsample with the full sample of the GSHS Argentina 2018, we found no age differences (mean age 14.8 years) but a higher preponderance of girls (65.9%) in the subsample compared with the full sample (52.0%). The gross enrolment in secondary schools in Argentina was 108% in 2019.^41^

In conclusion, psychosocial distress (anxiety-induced sleep disturbance, having no close friends and loneliness) increased the odds of MSA among both sexes. Interpersonal violence, low parental support and substance use among girls, and having multiple sexual partners among boys increased the odds of MSA. Furthermore, among both sexes, a higher number of psychosocial distress factors, social or environmental risk factors, and health risk behaviours increased the probability of MSA. Variables identified may potentially discriminate between MSA and SSA among adolescents in Argentina.

## Data Availability

The data generated during and/or analysed during the current study are available in the World Health Organization NCD Microdata Repository (https://extranet.who.int/ncdsmicrodata/index.php/catalog/).
